# Human thermal perception and time of day: A review

**DOI:** 10.1080/23328940.2021.1976004

**Published:** 2021-10-10

**Authors:** Marika Vellei, Giorgia Chinazzo, Kirsi-Marja Zitting, Jeffrey Hubbard

**Affiliations:** a Laboratory of Engineering Sciences for the Environment (LaSIE) (Umr Cnrs 7356), La Rochelle University, La Rochelle, France; b Department of Civil and Environmental Engineering, Northwestern University, Evanston, USA; c Division of Sleep and Circadian Disorders, Departments of Medicine and Neurology, Brigham and Women’s Hospital, Boston, MA, USA; d Division of Sleep Medicine, Harvard Medical School, Boston, Ma, USA; e Laboratory of Integrated Performance in Design (Lipid), School of Architecture, Civil and Environmental Engineering (Enac), École Polytechnique Fédérale De Lausanne (Epfl), Lausanne, Switzerland

**Keywords:** Circadian rhythms, light, time of day, diurnal, thermal perception, thermal comfort, experimental, observational

## Abstract

The circadian clock regulates diurnal variations in autonomic thermoregulatory processes such as core body temperature in humans. Thus, we might expect that similar daily fluctuations also characterize human thermal perception, the ultimate role of which is to drive thermoregulatory behaviors. In this paper, we explore this question by reviewing experimental and observational thermal comfort investigations which include the “*time of day*” variable. We found only 21 studies considering this factor, and not always as their primary analysis. Due to the paucity of studies and the lack of a specific focus on time-of-day effects, the results are difficult to compare and appear on the whole contradictory. However, we observe a tendency for individuals to prefer higher ambient temperatures in the early evening as compared to the rest of the day, a result in line with the physiological decrease of the core body temperature over the evening. By drawing from literature on the physiology of thermoregulation and circadian rhythms, we outline some potential explanations for the inconsistencies observed in the findings, including a potential major bias due to the intensity and spectrum of the selected light conditions, and provide recommendations for conducting future target studies in highly-controlled laboratory conditions. Such studies are strongly encouraged as confirmed variations of human thermal perceptions over the day would have enormous impact on building operations, thus on energy consumption and occupant comfort.

List of abbreviations: TSV: Thermal Sensation Vote; TCV: Thermal Comfort Vote; T_pref_: Preferred Temperature; TA: Indoor Air Temperature; RH: Indoor Relative Humidity; T_skin_: Skin Temperature; T_ty_: Tympanic Temperature; T_re_: Rectal Temperature; T_oral_: Oral Temperature

## Introduction

The central circadian clock in humans is responsible for diurnal fluctuations in the temperature thresholds regulating autonomic thermoregulatory responses [1–3]. These changes in thermoregulatory effectors in turn contribute to the modification of core body and distal skin temperatures [4–6] and affect sleep and waking behavior [7,8]. Thus, we could expect that similar circadian fluctuations might also affect thermal perception, the ultimate role of which is to drive thermoregulatory behavioral responses. From this perspective, a diurnal pattern in thermal perception and, in particular, a slight dip in core body temperature [4–6] accompanied by a shift in the preferred temperature toward a higher ambient temperature in the evening, might lead to warmth-seeking thermoregulatory behaviors, which are necessary for sleep preparation. This is corroborated through observations in other mammals [9].

Thermal comfort prescriptions given in international guidelines and standards do not take into consideration the “*time of day*” as a variable influencing acceptable indoor thermal conditions [10,11]. This assumption is primarily based on thermal comfort studies conducted in the 1970s and 1980s [12–17]. However, more recent evidence contradicts the findings from those early studies [18,19], suggesting that circadian variation plays a role in human thermal perception. The question of a time-varying thermal perception is neither exclusively theoretical nor limited solely to occupant indoor thermal comfort. In fact, time-varying indoor temperature set-points may help reduce energy use in buildings and promote their energy flexibility [20]. A dynamic indoor temperature might also contribute to a more healthy environment [21]. Taken together, these facts highlight the worthiness of this subject for investigation. However, very few studies exist on the topic, and most of them do not consider the “*time of day*” variable as the main influencing parameter in the evaluation of occupant thermal perception. A lack of a theoretical and multidisciplinary background involving notions of human physiology might explain this paucity of studies.

Here we aim to fill this gap by providing a comprehensive explanation about the physiology of thermal regulation and circadian timing. With these notions, the goal of the paper is to analyze the influence of the time of day on human thermal perception through the review of both observational and experimental thermal comfort studies. Specifically, we aim to: (1) clarify the existence (or absence) of diurnal patterns in human thermal perception, (2) provide an explanation for certain inconsistencies in the findings, and (3) discuss recommendations for conducting future targeted studies.

To our knowledge, no review paper to date has investigated the dependency of human thermal perception on the time of day, although some past reviews on thermal comfort have offered cursory overviews of the subject [20,22–25]. Specifically, Webb [22] and te Kulve [23] independently reviewed the effects of light exposure on thermophysiology, thermal comfort, and health, and given that photic input is the primary synchronizer of the circadian clock, also considered daily variations of thermophysiology and health. However, diurnal modifications of thermal perception were not reviewed. Mishra [20] conducted a thorough review of thermal comfort studies investigating the effects of various spatial and temporal non-uniformities, including a limited section about diurnal variations of thermal preference. Wang [24] and Schweiker [25] independently surveyed sources of diversity in human thermal perception and dedicated a paragraph to circadian rhythms related to variations in core body temperature. However, none of these works focused specifically on reviewing studies investigating the dependency of thermal perception on the time of day.

In section ‘Physiology of thermoregulation and the circadian clock’ of this review paper, we introduce the basic physiological processes related to thermoregulation and the circadian clock. In section‘Review of thermal comfort studies’, we summarize the findings from 21 experimental and observational investigations studying the “*time of day*” variable in the context of thermal comfort research. Based on the physiological evidence outlined in section ‘Physiology of thermoregulation and the circadian clock’, section ‘Discussion’ explores potential explanations for the inconsistencies observed in the findings and provide recommendations for conducting future studies that can address the existing knowledge gap.

## Physiology of thermoregulation and the circadian clock

### Thermoregulation

Humans, like nearly all mammals and avian species, are homeotherms (warm-blooded), meaning that their internal body temperature remains stable irrespective of environmental influence [26]. This is in contrast to poikilotherms (cold-blooded), such as fish, reptiles, and amphibians, whose internal temperature fluctuates widely depending on different factors, and can both influence metabolic rate and radically alter cellular processes (i.e. protein denaturing at high temperature) [27]. In the past, thermoregulation was thought to be exclusively under the control of the preoptic hypothalamus, a brain region located posterior to the optic nerve, and ventral to cortical structures [28]. Generally speaking, the hypothalamus is responsible for the regulation of a wide array of autonomic functions, including appetite and sleep initiation, and forms a critical part of the hypothalamic-pituitary-adrenal axis, which controls among others, stress reactions. In recent years, however, its role in regards to being the sole controller of thermoregulation in the brain has been redefined [29]. For example, thermoreceptors have been found in other areas, including the brain stem, and indeed in other central nervous system structures such as the spinal cord [30], underscoring the fact that the thermoregulatory system is diffuse [31].

Autonomic thermoregulatory processes manifest physiologically throughout the organism and consist primarily of vasodilation, vasoconstriction, sweating, and shivering, which depend on whether a person needs to increase or decrease their core body temperature in response to external stimuli, or indeed changes in immune function (e.g. infection-induced fever). Thresholds are defined as core body temperature values triggering each effective thermoregulatory defense at a given mean skin temperature. Thus, the regulated variable in the thermoregulation system is an integrative temperature signal, which incorporates spatially distributed core body and skin temperatures [32]. When a person is exposed to a hot environment (hyperthermia), the body reacts specifically to this condition through active cutaneous vasodilation and sweating [33,34]. In contrast, hypothermia is more difficult for the body to counteract, and in mild forms is manifested with cutaneous vasoconstriction and shivering. Interestingly, although the external environment is the most likely cause for the initiation of thermoregulatory processes, local changes in the hypothalamus can override any temperature modification at the extremities of the body, suggesting a tight coupling of brain and body temperature [35]. It should also be noted that a variety of aspects related to skin composition can influence temperature at the surface of the organism. For example, in humans, this is the case regarding skin type heterogeneity, specifically the amount of hair present. Indeed, an increase in hair follicle amount will augment the organism’s capacity to thermally insulate and increase the thermal stability of the skin [32].

The human thermoregulatory system has at its disposal also behavioral mechanisms, which are driven by the perceived thermal discomfort. The term “*thermal perception*” can refer to different semantic dimensions, such as sensation, preference, acceptability, dissatisfaction, comfort, pleasure, etc. The most objective or descriptive dimension is represented by thermal sensation (i.e. feeling warm, neutral, cold, etc.), which is typically assessed with the ASHRAE seven-point scale [10]. While thermal comfort is the affective or hedonic component of thermal perception and is thought to be important for activating behavioral thermoregulatory responses in humans, such as putting on/taking off clothing, changing activity level or posture, consuming hot/cold food or drinks, etc [36]. These thermoregulatory behaviors have been defined as “*an attempt to avoid what humans call thermal discomfort or displeasure and to obtain thermal pleasure*” [37]. Of note, the neural basis for thermal pleasure was recently described in an imaging study [38].

### Circadian rhythms

The circadian system, which is synchronized to the 24-hour day by signals from the environment (a process called entrainment), provides an adaptive mechanism for organisms to coordinate their physiological functions and behaviors with the Earth’s 24-hour light and dark cycle. This system consists of a central circadian clock and peripheral clocks present in every cell of the body [39]. The central circadian clock is located in the suprachiasmatic nucleus of the hypothalamus and in humans has an endogenous rhythmicity with a period slightly greater than 24 hours (hence the term circadian, i.e. “*circa diem*” or “*approximately 24 hours*”).

Light exposure, and in particular the light-dark cycle, is the primary synchronizer of the circadian clock to the 24-hour day, although sleep, physical activity, meal timing, and temperature can also synchronize daily rhythms [40,41]. Furthermore, the synchronizing effect of light helps the biological clock maintain a normal sleep-wake schedule (i.e. sleeping at night and being awake during the day). In addition to sleep, the circadian system coordinates rhythmicity in many of the body’s physiological functions, such as hormonal secretion, physical activity, and thermoregulation [42]. Circadian variation in these physiological functions can be experimentally observed in either “*constant routine*” or “*forced desynchrony*” laboratory protocols, which are the two gold standard methods for assessing circadian rhythmicity in humans. In constant routine protocols, the effects of the exogenous influences on circadian rhythms are controlled by keeping subjects awake in a semi-recumbent posture under constant and controlled conditions of light, temperature, and food and fluid intake [43,44]. In forced desynchrony protocols, subjects live on an imposed sleep-wake cycle with a period that is much shorter or longer than the endogenous circadian period for at least a week, which leads to the dissociation of the endogenous and activity-related rhythms [45,46]. For clarity, throughout the rest of this review, we will refer to “*circadian*” variation when discussing studies that were conducted under these two conditions and use “*daily”* or “*diurnal*” variation when discussing studies that were conducted under either real-life conditions or under controlled, but neither constant nor desynchronized (e.g. sleep is allowed at normal times), conditions [6].

#### Circadian control of body temperature

**Figure 1.**
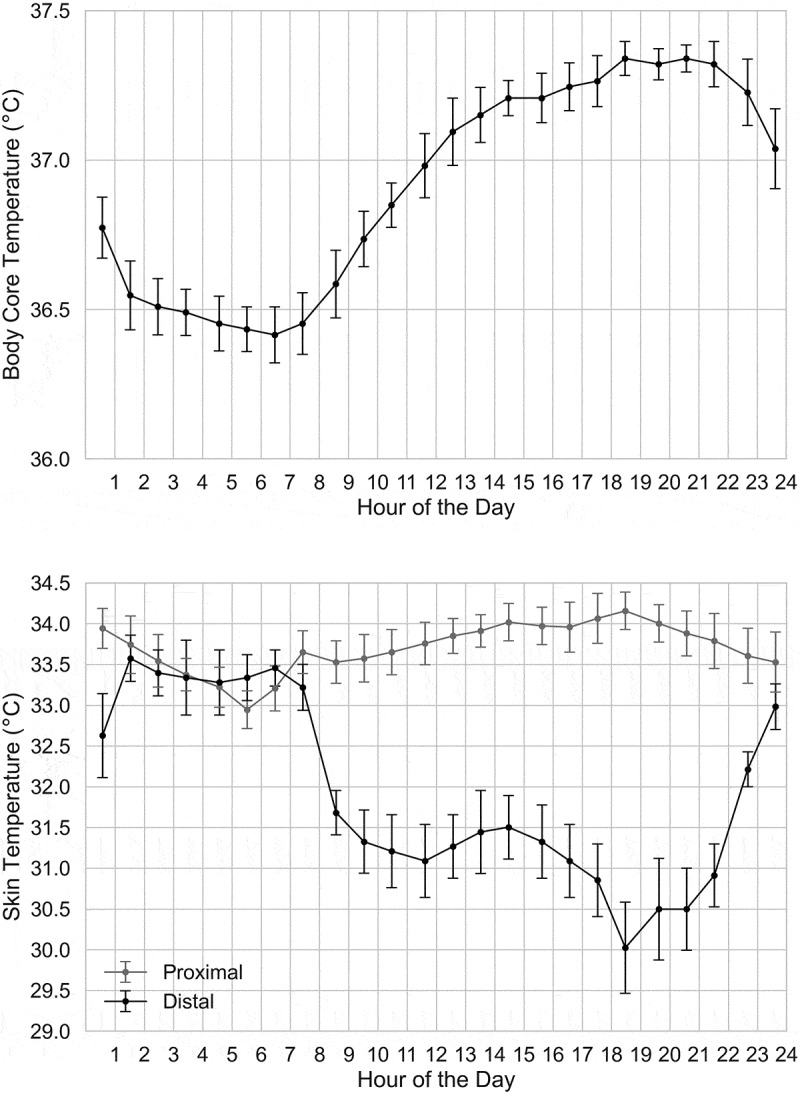
*Core body temperature (above) and proximal and distal skin temperatures (below) as a function of the time of day (hours, mean ± standard deviation). Adapted from Cuesta et al. (2017)* [6]

A lower core body temperature at night is a well-documented physiological phenomenon that was first published in 1842 [47]. Distal skin temperature (i.e., temperature of hands and feet) has also been reported to vary with the time of day [5,6,48,49]. The distal skin temperature rhythm is inverted and displays higher amplitudes compared to the core body temperature rhythm. Furthermore, the maximal value in distal skin temperature is reached shortly before the core body temperature minimum which is observed around 04:00 in the morning [4–6]. The diurnal rhythm of the proximal skin temperature (e.g., infraclavicular region, sternum) is more complex but, under real-life conditions, appears to vary in phase with the distal skin temperature rhythm [48,49]. The time-course of core body temperature and distal and proximal skin temperatures across the 24-hour day under a normal sleep-wake schedule, but at constant posture and controlled levels of activity, light exposure, and food intake is shown in Figure 1. Under such controlled conditions, the diurnal variation in proximal skin temperature follows more closely the rhythm of core body temperature [6].

The daily rhythm in core body and skin temperatures is due to the circadian variation in heat production and heat loss and, in particular, the nocturnal increase in heat loss from the extremities, which leads to an increase in distal skin temperatures [1,4,50]. However, due to the simultaneous decrease in cardiac output, this increase in heat loss is modest and, thus, the decline in core body temperature is also relatively slow. There is also circadian variation in heat production such that the fasted resting metabolic rate (also known as basal metabolic rate) is highest during the late afternoon and lowest during the late night [46]. With respect to heat loss, circadian variations in the autonomic thermoregulatory responses have also been observed. During the night, the internal temperature thresholds for sweating and vasodilation have been shown to be lower than during the day, while the differences observed between the morning and the afternoon are smaller [1–3,51].

#### Sleep onset

The circadian temperature cycle and the onset of sleep are strongly linked and appear to be mainly regulated in similar brain structures: the preoptic hypothalamus. Research into human sleep found that vasodilated distal skin regions, particularly the hands and feet, are the best predictors of sleep initiation [8]. As a practical example, a hot bath (inducing vasodilation) prior to, but not immediately before the sleep period, is known to help with its initiation [9,52]. In contrast, transitions from sleep to wakefulness are accompanied by vasoconstriction [53]. Furthermore, given that skin warming appears to promote sleep, the preference for slightly cold thermal conditions during the day may help to induce mild skin cooling and, hence, boost daytime alertness, wakefulness, and overall vigilance [53]. However, these considerations are only applicable for thermal conditions within the thermoneutral zone of vasomotor regulation.

#### The role of light

Light is the primary synchronizer of the central circadian clock and functions by inhibiting the release of melatonin, a sleep-promoting hormone produced by the pineal gland. Photosensitive retinal ganglion cells are nonvisual photoreceptors and are responsible for sending light information from the retina to the suprachiasmatic nucleus and the central clock. Melatonin secretion is normally high at night (in the dark) and reaches practically zero during daytime. Melatonin enhances heat loss by influencing peripheral vasodilatation [54,55], which results in modification of core body and distal skin temperatures and regulation of sleep [7,8].

The role of light as a synchronizer of the circadian system has been studied experimentally by either directly looking at endocrine modifications of the melatonin concentration itself [56,57] or by observing physiological changes of core body temperature, distal skin temperature and vasodilation [58]. Changes in cortisol levels and heart rate have been also investigated [58]. The effect of light on these modifications has been shown to depend on the time of day and on the duration, spectrum (short vs long wavelength) and intensity (bright vs dim illuminance) of the light exposure [57,58].

The effect of light on the circadian rhythm depends on the circadian phase, i.e. on the time of day at which the light exposure occurs [59]. In the evening and early night, bright light, and in particular short wavelength (blue) light, induces phase-delays of the circadian rhythm [56] and diminishes the decline in core body temperature and the increase in distal skin temperature [60]. In the late night and early morning, light exposure results in phase advances and anticipates the increase in core body temperature and the decline in distal skin temperature, while no effect of light exposure on core body and distal skin temperatures has been observed in the afternoon [58]. Even though the effect of light history has not yet been reliably characterized, daytime bright light may reduce the sensitivity to light in the evening or night [61].

The effect of the duration of the light exposure on circadian phase resetting has been shown to be non-linear and asymptotic for durations above ∼4 h. A brief exposure (<12 minutes) to very bright light (∼10,000 lux) can induce modification of the melatonin concentration more efficiently per minute of light exposure than longer durations [57,62]. Thus, the phase-resetting effect of light appears to occur very rapidly, especially for very bright light.

Exposure to short wavelength visible light (between 400 and 500 nm, i.e. blue and blue-enriched light) has a stronger influence on the circadian resetting than longer wavelength light [63–66]. The greatest melatonin suppressing effect has been observed at the wavelength of 424 nm [67].

The effect of light exposure intensity on circadian phase resetting also follows a non-linear relationship. In the early night and for an exposure duration of 6.5 h, a ∼100 lux of light generates half of the circadian phase delay resetting response compared to ∼9,000 lux [56]. Thus, the effect of light on circadian rhythms appears to occur even after small changes in ordinary light exposure which has considerable practical significance [56,60].

## Review of thermal comfort studies

In this section we review experimental and observational studies in the thermal comfort field which included the “*time of day*” factor in their analysis of thermal perception. The literature search was first conducted automatically in the Scopus scientific database between 1970 and 2021. The search terms related to thermal perception were: “*thermal sensation*” OR “*thermal comfort*” OR “*thermal preference*” OR “*thermal perception*” OR “*preferred temperature*” OR “*neutral temperature*” OR “*comfort temperature*” OR “*temperature preference*”, and those related to circadian rhythms were: “*circadian*” OR “*time of day*” OR “*diurnal rhythm*” OR “*daily rhythm*” OR “*diurnal change*” OR “*daily change*” OR “*diurnal variation*” OR “*daily variation*”. Furthermore, we included only studies with human subjects (“*human*” OR “*subject*” OR “*person*” OR “*student*” OR “*occupant*” OR “*worker*”) which were published in English in peer-reviewed conference proceedings or journals. The terms were searched in the title, abstract and keywords of the papers. The automatic bibliographic search resulted in 111 papers.

We then manually reviewed the papers and retained for further analysis only those that specifically focused on the human thermal perception’s dependency on the time of day. The majority of the excluded papers did not collect any thermal perception votes, such as thermal sensation, thermal comfort, thermal preference, and instead described daily variations in indoor and/or outdoor environmental conditions and the related calculation/simulation of thermal comfort indices. Some studies [68,69] measured diurnal variations in indoor temperatures and collected the associated thermal perception votes but did not explicitly analyze changes in thermal perception with respect to the “*time of day*” variable and were, thus, excluded. The paper by Ngarmpornprasert & Koetsinchai [70] focusing on diurnal changes in productivity was also excluded because it did not specifically focus on thermal perception.

After this manual screening process, we were left with 19 papers. The “*reference by reference*” method was then used to find two additional publications. A total of 21 papers were finally considered for review. The selected papers are either experimental or observational studies and are presented in two separate sections.

For clarity, and in the context of this paper, we define morning as the time between 06:00 and 12:00, afternoon as the time between 12:00 and 18:00, evening as the time between 18:00 and 00:00, and night as the time between 00:00 and 06:00. However, these definitions are not universally accepted and may vary depending on the country, season, etc.

### Experimental studies

We identified a total of 15 experimental studies investigating daily variations in thermal perception. These studies were conducted in thermally-controlled climate chambers and included only young participants (between 20 and 30 years old). They can be classified according to two main features:

- type of thermal exposure for the perceptual response, which can be either a localized thermal stimulus or a whole-body thermal exposure.
- type of thermal assessment, which can be either passive or active. In the passive assessment, which is the most often used one in current thermal comfort research, the subject is experiencing a certain thermal condition and is concurrently surveyed about its thermal sensation, thermal comfort, thermal preference, etc. In the active assessment, the subject is actively engaged in adjusting the temperature of the climate chamber according to his/her preference until the most comfortable temperature is reached.

The majority of the 15 reviewed studies investigated responses to whole-body thermal exposures, while only two studies addressed localized thermal stimuli [71,72]. The reviewed studies are equally distributed in terms of passive/active thermal assessment, with the most recent studies focusing on the active one. An overview of the reviewed studies is given in Table 1.

**Table 1.** Summary of experimental studies investigating human thermal perception at different times of the day. NS stands for statistically not significant

Ref.	No. Subjects M/F (age: mean±SD in years)	Type of thermal assessment	Time of exposure	Thermal condition	Lighting condition	Time of Effect Measurement	Thermal perception effects	Thermo- physiological effects
Nevins et al., 1966 [12]; Rohles, 1971 [13]	800/800 (18 to 24)	passive	14:00 to 17:00	160 combinations of TA & RH	constant at 1450 lux	14:30 to 17:00	TSV: NS	-
			19:00 to 22:00			19:30 to 22:00		
Fanger et al., 1974 [15]	8/8 (22.5 ± 2.5)	Active	09:00 to 12:00	TA: 25.6°C, RH: 50% (initial)	-	10:00 to 12:00	Tpref: NS	Tskin, Tre: Higher in the evening
			19:00 to 22:00			20:00 to 22:00		
Fanger et al., 1974 [14]	8/8 (23 ± 1.5)	Active	day	TA: 25.6°C, RH: 50% (initial)	-	day	Tpref: NS	Tskin, Tre: Lower in the night
			night			night		
Rohles, 1979 [16]	54/54	passive	09:00 to 12:00	TA: 20.0, 25.6, and 31.7°C, RH: 50%	-	09:00 to 12:00	TSV: NS, TCV: NS	-
			13:00 to 16:00			13:00 to 16:00		
			18:00 to 21:00			18:00 to 21:00		
Attia et al., 1980 [71]	4	passive	00:00 to 02:00	TA: 25°C, RH: 45%	-	00:00 to 02:00	Tpref: Lower in the afternoon (15:00 to 17:00)	Tre: Higher in the afternoon (15:00 to 17:00)
			03:00 to 05:00			03:00 to 05:00		
			06:00 to 08:00			06:00 to 08:00		
			09:00 to 11:00			09:00 to 11:00		
			12:00 to 14:00			12:00 to 14:00		
			15:00 to 17:00			15:00 to 17:00		
			18:00 to 20:00			18:00 to 20:00		
			21:00 to 23:00			21:00 to 23:00		
Enander, 1982 [72]	18/0 (29.9)	passive	09:00 to 11:00	TA (hand): 0, 7, and 15°C	-	09:30 to 11:00	TSV: Lower in the mid-afternoon (15:00 to 16:30)	Tskin: Lower in the mid-afternoon (15:00 to 16:30)
			12:30 to 14:30			13:00 to 14:30		
			14:30 to 16:30			15:00 to 16:30		
Terai et al., 1985 [17]	23/0 (21.9 ± 3.3)	Active	07:00 to 08:00	TA: 31°C, RH: 40% (initial)	-	07:00 to 08:00	Tpref: NS	Tre, Tty: Higher in the afternoon (17:00 to 18:00)
			10:00 to 11:00			10:00 to 11:00		
			17:00 to 18:00			17:00 to 18:00		
			21:00 to 22:00			21:00 to 22:00		
Grivel & Candas, 1991 [73]	24/24 (22 ± 1)	Active	09:00 to 12:00	TA: 25.1°C, RH: 50% (initial)	-	11:00 to 12:00	Tpref: Higher in the late afternoon (17:00 to 18:00)	
			15:00 to 18:00			17:00 to 18:00		
Shoemaker & Refinetti, 1996 [75]	16/16	Active	15:00 to 17:00	TA: 25°C (initial)	-	15:00 to 17:00	Tpref: Higher at night (03:00 to 05:00) for male subjects	Toral: Lower in the night (03:00 to 05:00)
			03:00 to 05:00			03:00 to 05:00		
Kim & Tokura, 1997 [74]	0/7 (23.6 ± 2.5)	Active	06:30 to 09:00	TA: 23°C, RH: 60% (initial)	-	08:30 to 09:00	Tpref: Higher in the morning (08:30 to 09:00)	-
			19:30 to 22:00			21:30 to 22:00		
Shido et al., 2001 [76]	7/0 (22)	Active	09:00 to 11:00	TA: 26°C, RH: 60% (initial)	-	10:40 to 11:00	Tpref: Lower in the afternoon (15:40 to 16:00) for heat acclimated subjects	Tskin: NS, Tre: Higher in the afternoon
			14:00 to 16:00			15:40 to 16:00		
Kakitsuba & White, 2013 [77]	10/0 (22.2 ± 1.0)	passive	9:00 to 18:00	TA: 26 to 30°C, 28°C, 30 to 26°C RH: 60%	constant at 500 lux	9:00 to 10:00	Tpref: Higher in the late afternoon (17:00 to 18:00)	Tskin,Tre: NS
						13:00 to 14:00		
						15:00 to 16:00		
						17:00 to 18:00		
Kakitsuba, 2019 [78]	0/8 (20.8 ± 1.5)	passive	9:00 to 18:00	TA: 26 to 30°C, 28°C, 30 to 26°C RH: 60%	constant at 500 lux	9:00 to 10:00	Tpref: NS	Tskin: NS, Tty: Higher in the late afternoon (17:00 to 18:00)
						13:00 to 14:00		
						15:00 to 16:00		
						17:00 to 18:00		
Kakitsuba, 2020 [18]	13/13 (21.7 ± 0.8)	passive	9:00 to 18:30	TA: 28°C, RH: 40, 50, 60, 70, and 80%	-	9:30 to 10:30	TSV: Lower in the evening (17:30 to 18:30) for male subjects, TCV: NS	Tskin,Tty: NS
						13:30 to 14:30		
						17:30 to 18:30		
Kakitsuba, 2021 [19]	7/0 (22.1 ± 0.6)	passive	9:00 to 18:30	TA: 28°C, RH: 60, 70, and 80%	-	9:30 to 10:30	TSV: Lower in the evening (17:30 to 18:30), TCV: NS	Tskin,Tty: NS
						13:30 to 14:30		
						17:30 to 18:30		

#### Study by study description

In the early 1970s, Nevins and Rohles conducted a series of notable experiments exposing 1,600 subjects to 20 different air temperatures (ranging from 15.5 to 36.5°C with increments of 0.56°C) at each of eight different levels of relative humidity (15, 25, 35, 45, 55, 65, 75 and 85%) for three-hour periods [12,13]. A total of 160 different thermal combinations were tested and no subject was used for more than one test. Half of the students were tested in the afternoon (14:00 to 17:00) and the other half in the evening (19:00 to 22:00). Subjects were surveyed every half hour using a scale similar to the 7-point ASHRAE thermal sensation scale but the “*neutral*” vote was substituted with the “*comfortable*” vote. Over the three-hour test period and across all the different temperature levels, subjects’ thermal sensation was found to decrease, and the authors refer to this observed phenomenon as “*adaptation*”. No difference in thermal sensation was found between the afternoon and the evening tests. In a second series of experiment, Rohles exposed a total of 108 subjects to three different steady-state temperature conditions (20.0, 25.6, and 31.7°C) during the morning (09:00 to 12:00), afternoon (13:00 to 16:00), and evening (18:00 to 21:00) and both during a cold winter day and a warm summer day [16]. Again, the time of day was not found to affect participants’ thermal sensation response.

Compared to the studies by Nevins and Rohles, Fanger and colleagues used a smaller sample size (16 subjects) and an active thermal assessment method consisting of adjusting the temperature according to the preference of the subject until the most comfortable temperature was reached [15]. The room-temperature controller was not directly operated by the subject who could indicate every 10 minutes his/her thermal preference (warmer, colder, no change) to the experimenter in charge of the controller. Each subject participated in two tests in the morning (09:00 to 12:00) and two tests in the evening (19:00 to 22:00). At the start of each test the ambient temperature and relative humidity were set to 25.6°C and 50%. Mean preferred temperatures were found to be slightly lower in the evening, but the difference (0.2°C) was not significant. Fanger and colleagues conducted another similar experiment during the late afternoon and late night (i.e. at predetermined times when the subjects’ core body temperature was expected to be at maximum and minimum, respectively) and again found no significant difference in the mean preferred temperatures [14].

Attia and colleagues exposed four subjects to a constant room temperature of 25°C and relative humidity of 45% at eight different times of the day [71]. For the first 30 minutes of the exposure the subjects were sitting and were then performing bicycle ergometer work at 50 W for the remaining 45 minutes. Rectal temperature (i.e. core body temperature), skin temperature and onset of sweating were measured. The most pleasant temperature on the back of the neck (localized thermal stimulus) was chosen by voluntary control (i.e. the subject was able to control the temperature of the localized stimulus and choose the temperature they considered to be the most pleasant) at the end of the 30 minutes sitting period and was found to be lowest in the afternoon (15:00 to 17:00).

Enander exposed the hand of 18 males to air temperatures of 0, 7 and 15°C for 95 minutes at a time at three different times of the day: morning (09:00 to 11:00), early afternoon (12:30 to 14:30), and mid-afternoon (14:30 to 16:30). Subjects reported feeling the coldest in the mid-afternoon, particularly at 7°C [72].

Terai and colleagues studied the preferred temperature in 23 male students, who were tested at four different times of the day (at 07:00, 10:00, 17:00 and 21:00). The students were then able to adjust the temperature by a remote controller for the next 40–50 minutes until the most comfortable temperature was reached [17]. At the start of each test the ambient temperature and relative humidity were set to 31°C and 40%. Rectal temperature, tympanic temperature, mean skin temperature, heart rate and O_2_ consumption and CO_2_ production were recorded. The lowest mean preferred temperature was found at 17:00, but the difference was not statistically significant. However, one third of the subjects operated the room-temperature controller frequently in both upward and downward directions and consequently had difficulties in reaching thermal equilibrium.

Grivel & Candas used the same method as Terai and colleagues but had a larger sample size consisting of 48 young adults [73]. The experiments were done both during the morning (09:00 to 12:00) and the afternoon (15:00 to 18:00). At the start of each test the ambient temperature and relative humidity were set to 31°C and 40%, and rectal and mean skin temperatures were continuously recorded. Only data from the third stable hour of each session were considered for the analysis as the first hour was needed to stabilize core body temperature and the second hour for finding the most comfortable temperature. Mean preferred temperatures were found to be highest between 17:00 and 18:00, and the difference (1.5°C) was significant.

Kim & Tokura used the same active thermal assessment method with seven young female subjects tested in the morning (06:30 to 09:00) and in the evening (19:30 to 22:00) [74]. At the start of each test the ambient temperature and relative humidity were set to 25.1°C and 50%. Only the last 30 minutes of data from each session were included for analysis. The subjects preferred significantly higher room temperatures in the morning than in the evening.

Shoemaker & Refinetti investigated the upper and lower temperature thresholds for discomfort in 32 undergraduate students both during the afternoon (15:00 to 17:00) and at night (03:00 to 05:00) [75]. For half of the subjects the temperature was raised during the experiment, while for the other half the temperature was lowered with a rate of temperature change equal to 0.7°C/min and starting from an initial temperature of 25°C. For male subjects, the temperature thresholds at which the subjects began to feel thermally uncomfortable were found to be significantly lower in the afternoon than at night, while for female subjects the difference was not significant.

Shido and colleagues investigated preferred ambient temperatures (which could be changed by turning a knob) in seven heat-acclimated and non-heat-acclimated subjects during the morning (09:00 to 11:00) and afternoon (14:00 to 16:00) [76]. The heat-acclimated subjects were previously exposed to heat only in the afternoon. Subjects’ rectal and skin temperatures and O_2_ consumption and CO_2_ production were recorded. The preferred self-selected ambient temperature in the afternoon was significantly lower than that of the morning in heat-acclimated subjects. There was no difference in preferred temperature in the non-heat-acclimated subjects.

Kakitsuba continuously exposed ten Japanese male subjects [77] and eight Japanese female subjects [78] to three different air temperature conditions characterized by stepwise increases from 26 to 30°C, steady at 28°C and stepwise decreases from 30 to 26°C between 9:00 and 18:00. Relative humidity was controlled at 60% under all conditions. Rectal/tympanic temperature, mean skin temperature, local heat flux rates, O_2_ consumption and CO_2_ production, thermal sensation and thermal comfort were monitored throughout four time periods: morning (9:00 to 10:00), early afternoon (13:00 to 14:00), mid-afternoon (15:00 to 16:00), and late afternoon (17:00 to 18:00). Male (but not female) subjects were more comfortable with higher temperatures in the late afternoon.

In a first series of winter experiments, Kakitsuba exposed 26 lightly clothed Japanese men and women between 9:30 and 18:30 to different steady-state thermal conditions characterized by an air temperature of 28°C and five relative humidity levels: 40, 50, 60, 70, and 80% [18]. Tympanic temperature, mean skin temperature, local heat flux rates, O_2_ consumption and CO_2_ production, thermal sensation and thermal comfort were monitored throughout three periods: morning (9:30 to 10:30), afternoon (13:30 to 14:30), and evening (17:30 to 18:30). No significant differences in tympanic temperature, mean skin temperature and thermal comfort were observed between the three periods under any of the conditions but significant effects of the time period on the thermal sensation vote were observed for the male (but not for the female) subjects who felt coldest in the evening. Kakitsuba conducted another experiment with the same experimental protocol but conducted in summer with seven lightly clothed Japanese male subjects exposed between 9:30 and 18:30 to different constant thermal conditions characterized by an air temperature of 28°C and three relative humidity levels: 60, 70, and 80% [19]. Stratum corneum water content and finger blood flow rate were monitored together with tympanic temperature, mean skin temperature, thermal sensation and thermal comfort during three periods: morning (9:30 to 10:30), afternoon (13:30 to 14:30), and evening (17:30 to 18:30). As in the winter study, the male subjects’ thermal sensation was found to be lowest in the evening and the difference compared to morning and afternoon was significant.

#### Overview of the results

Among the 15 reviewed experimental studies investigating diurnal variations in thermal perception, six found no significant differences in thermal perception [12–17,78] six observed significant differences [19,71–74,77], two observed significant differences but were conducted only with male subjects [18,75] and the remaining one observed significant differences only for heat-acclimatized subjects [76]. In addition, two out of the six studies finding significant differences observed these differences only in male subjects [19,72]. Therefore, it could be argued that variations are generally more evident in males than females.

**Figure 2.**
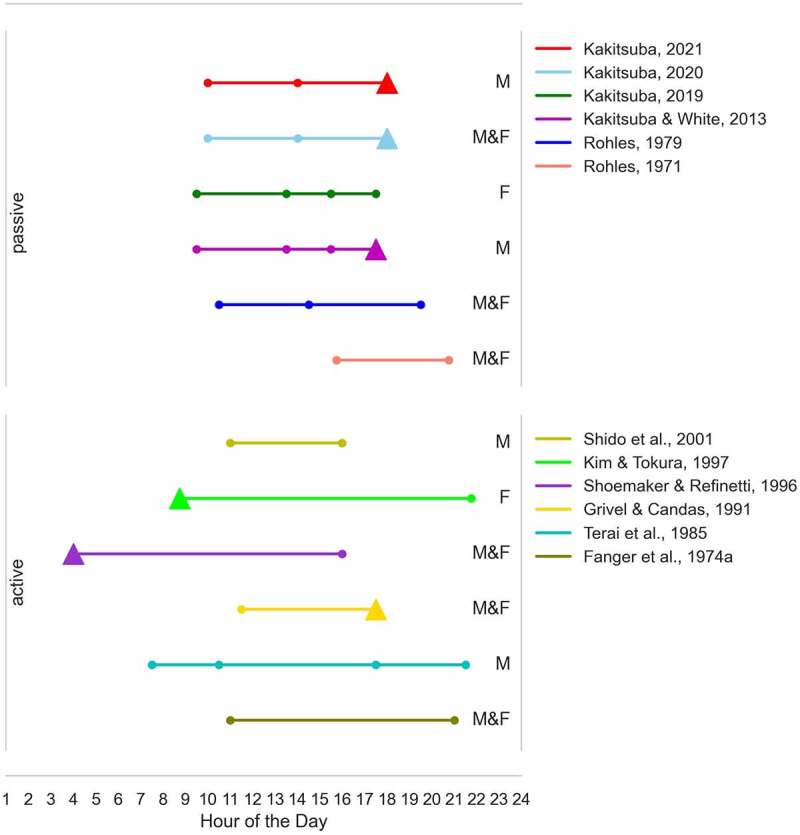
Overview of the results from the experimental studies. Only studies investigating thermal perceptual responses to whole-body thermal exposures are reported. The investigated hours are indicated with the points. The triangles indicate an hour of the day during which a preference for significantly higher temperatures with respect to the other investigated hours was observed. M indicates male subjects, while F indicates female subjects

An overview of the results of studies investigating thermal perceptual responses to whole-body thermal exposures is given in Figure 2. The reported studies are ordered in chronological order (with the newest on top) and grouped according to the type of thermal assessment (active vs. passive). In Figure 2, we do not report the size of the observed effect but only the direction of the significant effect. It is challenging to compare the effect sizes because of the different thermal assessment methods. As highlighted in Figure 2, based on the results from four studies [18,19,73,77], there is a tendency to prefer higher temperatures in the early evening compared to other times of the day. However, the results from at least four other studies [12–17] seem to contradict these results.

### Observational studies

Only six studies have investigated diurnal variation in thermal perception in field conditions. All these studies were observational, meaning that the thermal conditions were not controlled unlike in the experimental investigations, but were only observed and recorded. The considered studies can be divided into two main groups:

- Studies investigating thermal preferences of participants by observing the chosen temperature set-points in buildings at various times of the day (no subjective data collected);
- Studies comparing thermal perception of participants (data collected with subjective questionnaires) at different times of the day at constant temperatures or in response to varying temperatures. In the latter case, these temperature variations are recorded and then correlated to participants’ thermal perception e.g., by binning similar temperatures together or by assessing the neutral temperature.

These observational studies differ in terms of the number and characteristics of the buildings and the participants that were considered, the season in which the investigation was conducted and the type of data that was collected (both environmental and perceptual). In addition, in most of the investigations, the analysis of the diurnal variation of the temperature profiles or thermal perception was not the primary goal of the study. The “*time of day*” variable was recorded and analyzed among many other factors affecting the thermal perception of the participants. An overview of the reviewed studies is given in Table 2.

**Table 2.** Summary of observational studies investigating the effect of the time of day on human thermal perception

Ref.	Number of households (subjects)	Thermal measurement	Subjective data	Time of the year	Building type	Space type	Lighting condition	Temperature profile/thermal perception change
Vellei et al. (2021) [84]	10,000	From smart heating controller	-	All year round	Home	-	-	Temperature set-point higher in the evening
	(7,000 subjects)	TA + RH + Air velocity	TSV	All year round	Both residential and office buildings	-	-	Neutral temperature higher in the evening
Hanmer et al. (2019) [79]	71	From smart heating controller	-	Winter (January and February)	Home	Living room	-	Temperature set-point higher in the evening (peak at 20:00)
Huebner et al. (2015) [80]	275	TA	-	Winter (mid-July 2007 to early February 2008)	Home (Terraced, Semi-detached, Bungalow or detached house, Flats)	Bedroom and living room	-	Two-peak (40%) Flat line (30.9%) Steady rise (15.3%) Steep rise (13.8%)
Karyono (2000) [82]	7 (596 subjects)	TA	TSV	-	Multi-storey office buildings	Office	-	Neutral temperature higher in the afternoon (between 15:00 and 16:00) than in the morning (between 09:00 and 10:00)
Pollmann (1994) [83]	1 (24 subjects)	Constant TA	TSV (10-point scale)	-	Factory	-	Artificial light (not measured)	Constant temperature (23.4 ± 0.45°C) perceived warmer in the late afternoon compared to the rest of the day. Same temperature is perceived colder in the evening and night.
Wagner at al. (2007) [81]	1 (50 subjects)	TA + RH	TSV	Summer (4 weeks in 2005)	Office	Office	-	TP higher in the afternoons (14:00–16:00) than in the mornings (8:00 to 10:00)

#### Study by study description

Two studies [79,80] analyzed daily variation in thermal preference by observing temperature setpoint variations in UK households. Both studies highlight a general preference toward warmer temperatures in the evening compared to the morning.

Hanmer and colleagues investigated 71 UK households’ reactions to variations in the daily temperature profile resulting from a change of heating system: from conventional boiler heating to hybrid pump (i.e., air source heat pump in combination with a gas boiler) [79]. This change implies a change in pre-heating time and in the resulting temperature distribution over the day. With the new system, the pre-heating phase takes place before the occupants have requested warmth, unlike with conventional boiler heating. The analysis considered the recordings of the temperature values (measured from the thermostat), the temperature setpoint chosen by the user and the outside temperature at a local weather station recorded over 2,096 days in January and February for the 71 households. Together with environmental measurements, interviews were conducted in 11 households before and after the change of the heating system. The recorded data from households with the hybrid pump was compared with data from a database that included information from 3,579 homes with conventional gas or oil boilers. Results indicated higher indoor temperatures in the households with the hybrid pump, compared to those recorded in households with conventional boilers. This increase in daytime and evening temperatures in the building was welcomed by three of the 11 households, while four of the 11 households complained of increased nighttime temperature in the building. The results also showed that many households’ temperature preference varies across the day: two higher temperature setpoints were observed, one in the morning (8:00) and the other in the evening (20:00), with the evening peak in temperature being higher than the morning peak. Warmer temperatures were preferred in the evening compared to the rest of the day as evidenced by the households changing the thermostat settings to a higher level over the course of the day. Based on these interviews, the authors argued that such changes in temperature preferences may be due to different activity levels at different times of day (i.e., more sedentary in the evening compared to the rest of the day) and occupancy patterns (i.e., the household is primarily occupied in the morning and in the evening). However, the authors additionally point out that this does not explain why some of the interviewed people who spent their day working from home, a relatively sedentary activity, did not turn on their heating system at all. A direct connection between activity levels and interactions with the heating system has therefore not been identified in the study.

Huebner and colleagues investigated how temperature patterns varied over the course of a day (i.e., temperature profiles) in 275 UK homes over a three-month period in winter [80]. For this purpose, they performed a cluster analysis of the measured temperatures to detect different temperature profiles. They also investigated whether different temperature profiles can be related to socio-demographic variables such as age, income, tenure and household size, and to building-demographic variables such as type of housing and heating system. Their results highlighted the presence of four temperature profiles: two profiles with lower temperatures in the morning (6:00–7:30) and higher temperatures in the evening (21:00–23:30), one flat profile and one a “double-peak” profile (morning and evening peaks), with a higher peak in the evening (21:00–23:30). What was always observed was a trend of decreasing temperatures during the night (from 00:00 to around 7:00). The different temperature profiles were associated with variations in age and income of the inhabitants as well as type of housing.

Three other studies [81–83] investigated the effect of the time of day on thermal perception using subjective questionnaires administered at least twice during the day. In addition to the questionnaires, environmental variables were collected across the day.

Wagner and colleagues conducted a survey on 50 subjects working in a naturally ventilated office building in Karlsruhe, Germany, to investigate their thermal sensation responses [81]. Among several analyses conducted by the authors, they also investigated the different responses across the day (morning vs. afternoon) over four weeks. They studied the distribution of indoor operative temperatures with respect to the thermal sensation responses in the morning (8:00–10:00) and in the afternoon (14:00–16:00). Their results show that, on average, people prefer higher temperatures in the afternoon compared to the morning. For example, subjects expressed they felt “*just right*” at about 24°C in the morning and at about 25°C in the afternoon. Similarly, they indicated feeling slightly warm at about 25°C in the morning and at about 26°C in the afternoon. The other thermal sensation categories were either not reported by the participants or did not include enough responses to be compared between the two time periods.

Karyono conducted a field study in seven multi-storey office buildings in Jakarta, Indonesia, to investigate the thermal comfort of people in terms of neutral temperature [82]. The author investigated the thermal comfort of participants according to several individual characteristics including the sex, age, body mass index, and ethnic background of the participant, as well as according to the time of day. The results from the time-of-day analysis showed that neutral temperature for participants was, on average, higher in the afternoon (between 15:00 and 16:00) than in the morning (between 09:00 and 10:00). The difference between morning and afternoon neutral temperatures was 4°C, in terms of air temperature, and 3.1°C in terms of operative temperature. These results highlight again a preference for higher temperatures in the afternoon compared to the morning.

Pollmann investigated the circadian and circannual variations in thermal comfort in 24 male workers in an industrial setting [83]. The study was conducted in a controlled environment (temperature at 23.4 ± 0.45°C and relative humidity between 25 and 35%) and included the measurement of the thermal comfort of workers on a ten-point scale every hour during a morning, evening and night shift (starting at 6:00, 14:00 and 23:00 respectively). Results of the study showed that this temperature was generally considered as warm (above “*5*” on the ten-point scale), but it was reported to be perceived warmer in the late afternoon (around 17:00–18:00) compared to the rest of the day. Such increase of thermal perception observed over the afternoon (reaching the peak previously described) and its decrease during the evening and night shifts suggests that the constant temperature was perceived colder in the evening and night compared to the rest of the day.

Finally, Vellei’s more recent study [84] analyzed daily variations in temperature preference by using both temperature setpoint data from about 10,000 Canadian households and 22,000 samples of complete (objective + subjective) thermal comfort field data from about 7,000 subjects included in the ASHRAE Global Thermal Comfort Database I (ASHRAE I) plus the European Smart Controls and Thermal Comfort (SCATs) dataset. Thus, Vellei’s study considered a much larger sample than the previous works and was specifically dedicated to analyzing the “*time of day*” factor in the context of thermal perception. Results of the analysis of the temperature setpoint data showed that, at equal indoor air temperature and for occupied hours, when the occupants decided to change their setpoint temperature, they were mostly decreasing it in the middle of the day, while increasing it in the morning and early evening. Then, with the help of the ASHRAE I and SCATs datasets, it was further showed that the neutral temperatures have a daily trend with a minimum at around 14:00, and increase progressively in the rest of the day, indistinctively in the morning and evening. Vellei estimated that the neutral temperature differences between 08:00 and 14:00 and 14:00 and 20:00 is of the order of 2°C.

#### Overview of the results

The temperature set-point variation analysis presented in three of the six studies [79,80] showed that, in normal settings, people predominantly prefer higher temperatures in the evening compared to other times of the day. Despite the morning peak in the temperature set-point that was observed in some of the studies, the morning peak was always smaller than the evening peak. Another common feature in all the temperature profiles was the decrease in temperature at night (after 00:00). The lower temperature at night and the increase in activity in the morning may underlie the observed morning peak in temperature, whereas the consistent evening peak in temperature may be associated with both participants’ activities and with physiological changes. The observed variations in set-point temperature profiles and absolute temperatures could be due to inter-individual differences between participants as well as differences in the buildings that were analyzed, as highlighted by the results from Huebner and colleagues [80].

The studies investigating participants’ thermal perception through questionnaires also reported a general preference for warmer temperatures later in the day compared to the morning. More specifically, the studies of Karyono and Wagner which compared neutral temperatures and thermal sensation votes from the morning (8:00–10:00) with those from the afternoon (14:00–16:00), concluded that people preferred warmer temperatures in the afternoon than in the morning. Pollmann’s results appear to be in contrast with this conclusion as the participants in his study indicated that the constant temperature to which they were exposed to for a day was warmer in the late afternoon compared to the rest of the day, implying that people prefer a colder temperature in the afternoons. A decrease in thermal sensation is reported in the evening and at night, after the afternoon peak in thermal perception. Similarly, Vellei observed that the neutral temperature is higher in the evening and night compared to the afternoon. These results corroborate the hypothesis that a warmer temperature is preferred in the evening and at night compared to the rest of the day, as reported in the field studies investigating daily temperature profiles. The contrasting results among the four field studies investigating subjective responses with respect to thermal preference in the afternoon may be due to differences in thermal perception evaluation and data analysis, in the investigated populations and/or in different experimental design (including overlooked differences in metabolic rates due to varying levels of activities during the day). For example, Karyono estimated neutral temperature without considering the other thermal variables and by using a method (the Griffiths method), which has been shown unreliable [85,86], and Pollmann’s study was conducted in a factory environment characterized by a fixed-temperature, but the other thermal comfort variables were not reported and might have varied throughout the day. The limited number of studies and the different experimental methods used make direct comparison between observational studies challenging.

## Discussion

Among the 15 reviewed experimental studies investigating diurnal variations in thermal perception, six found no significant differences in thermal perception with the time of day while nine found some significant differences. A similar contradictory panorama is provided by the six observational studies included in this review. In this Section we focus on the results from the experimental studies and list some of the possible sources of bias for the observed inconsistencies. While we recognize that many factors could influence thermal preferences (e.g., several individual differences including acclimatization, and sensitivity to temperature) in this section we analyze the principal factors that could be responsible for the observed inconsistencies in the experimental studies considered. We also discuss some recommendations for conducting future targeted studies that could help the research community to better understand the role of the time of day on human thermal perception.

### Individual differences in circadian timing

Inter-individual differences in circadian phase and chronotype (i.e. the behavioral expression of individual circadian rhythmicity) are due to both endogenous differences in the circadian period and exogenous differences in the exposure to the external factors which can shift an individual’s circadian rhythms [40]. These differences can make it difficult to detect time-of-day effects on thermal perception. However, many of the exogenous differences such as those caused by shift work, irregular sleeping and eating patterns, jetlag, and irregular light exposure patterns, can be controlled for, which was not done in the reviewed studies. For example, shift workers and those with irregular schedules should be excluded from the study as should be those individuals who have traveled across several time zones in the weeks or months prior to the study. Moreover, in order to minimize the influence of individual differences, subjects should follow a stable

sleep-wake schedule (i.e. go to bed/turn off the lights and get out of bed at the same time each day) for at least a week before the start of the study in order to ensure that they are stably entrained to the local environment and to their individual sleep-wake schedule.

### Controlling for light

Light is likely the most important source of bias in the experimental and observational studies conducted so far. In the previous sections we have highlighted that the role of light as a circadian synchronizer strongly depends on the circadian phase, i.e. on the time of day at which the light exposure occurs, and is characterized by non-linearity with respect to the duration, spectrum, and intensity of the light exposure. In particular, in the transition periods, i.e. morning and evening, the effect of light on circadian resetting appears to occur even after brief exposures and small changes in light exposure [56,57,60].

However, only three out of the 15 reviewed experimental studies reported the details of the light exposure and only in terms of intensity (lux) [12,16,77,78]. None of the reviewed observational studies measured any characteristics of the light exposure. In the experimental studies of Kakitsuba & White [77] and Kakitsuba [78], light was controlled at a fixed value of 500 lux. This is in contrast to the chamber experiments of Nevins and Rohles [12,13], which were conducted during three hours at a controlled fixed high value of illuminance measured at desk height where the subjects were seated at approximately 135 foot-candles (1450 lux). For experiments taking place in the evening, this bright illuminance may have delayed the circadian rhythms, reduced the changes in core body and distal skin temperatures [23,67], and consequently, also changed the subjects’ thermal perception.

Lux is a measurement of the luminous flux weighted according to a “luminosity function” which depends on the human eye’s sensitivity to the different wavelengths, and it is thus considered to be perception at the level of the human eye. However, as previously stated, nonvisual photoreceptors in the retina, which are responsible for modulating circadian phase through circadian resetting, are most sensitive to shorter wavelengths of light (<480 nm). Of note, all the experimental studies were conducted in artificially illuminated climate chambers likely resulting in inconsistent spectral profiles. Therefore, the results of these studies could have been systematically compromised by the spectral effects of light, but this cannot be confirmed given that these characteristics were not reported.

Therefore, in future experimental studies on thermal perception, the effect of light should be systematically controlled and the exposure characteristics sufficiently detailed, as part of the experimental design. This should be done in terms of both intensity and spectrum, and light exposure in the evenings and mornings, especially to short wavelength blue and blue-enriched light, should be avoided because this could compromise the measurements of diurnal variations in thermal perception by phase-shifting the circadian clock, either by advancing (in the morning) and/or by delaying (in the evening) it.

### Controlling for the menstrual phase in fertile women

As we discussed in section ‘Review of thermal comfort studies’, the effect of circadian rhythms on thermal perception is generally more evident for male than female subjects. Past thermal comfort studies, including some of those reviewed here [12–17], already highlighted that variance in the thermal sensory response is greater in female versus male subjects. This has been attributed to several factors, including the effect of the menstrual cycle. The menstrual cycle in fertile women causes regular fluctuations in core body and skin temperatures with a period of approximately 28 days. This is due to the varying levels of estrogen and progesterone which cause shifts in all autonomic thermoregulatory thresholds (shivering, sweating and cutaneous vasodilation) [87]. As a result, both core body and skin temperatures are higher in the luteal phase compared with the follicular phase [49]. Indeed, Shoemaker & Refinetti [75] and Kim & Tokura [74] found significant differences in thermal perception associated with the time since the last menstruation and the luteal/follicular phase, respectively. It therefore seems that, depending on where women are in their menstrual cycle, the effect of time of day on thermal perception may be at least partially masked and, thus, menstrual cycle should be a controlled variable in future experimental and observational studies.

### Studying thermal conditions other than neutrality

As we have seen in the section ‘Review of thermal comfort studies’ the reviewed experimental studies have adopted two different types of thermal assessment, either passive or active. Nevertheless, the majority of the studies on thermal conditions were conducted in thermoneutral conditions. Even in studies where the subjects could actively adjust the temperature to reach a preferred value (active thermal assessment), the initial thermal conditions were always in the proximity of thermal neutrality. The only exceptions were the experiments of Nevins and Rohles [12,13], Shoemaker & Refinetti [75], Kakitsuba & White [77] and Kakitsuba [18,19,78]. Thus, diurnal variations in the cold and warm sensation thresholds were mostly disregarded. It could be that diurnal variations in thermal perception are more important at the extremes, affecting sensitivity to warm and cold stimuli at certain times of the day, while having little effect near the center of thermoneutrality. Sensory functions other than temperature perception have been observed to have diurnal variations in their recognition thresholds. For example, the salt recognition threshold was found to exhibit a circadian variation with the lowest values recorded in the afternoon [88]. In addition, the recognition thresholds for sweet compounds also showed a daily variation [89]. Therefore, future experimental studies should be focused on studying greater variations in thermal conditions and should try to identify whether warm and cold sensation thresholds are affected by the time of day.

### *Focusing on the “*transition*” periods*

By looking at Figure 1 showing the diurnal pattern of distal skin temperature in a controlled but not constant (i.e. sleep is allowed) condition, we can observe that most of the temperature variation takes place after waking up in the morning and before going to sleep in the evening. In fact, in the early to mid-morning (06:00 to 10:00) and in the evening (18:00 to 22:00), humans experience a state of transition [90], which is a “*heat gain*” in the morning (as the core body temperature climbs) and a “*heat loss*” in the evening (as the core body temperature drops). We refer to these periods, which are strongly related to the sleep/wake (rest/activity) state, as “*transition*” periods. Similarly, the influence of light on thermophysiology has been mainly observed in the morning and evening, while in the afternoon no effect of light exposure on core body and distal skin temperatures was found [58]. Therefore, while future experiments should collect data from across the 24-hour day, it seems particularly important to focus on these transition periods, morning and evening, and to compare them with the middle of the day where relatively little seems to happen in terms of circadian thermo-physiological variations.

### Distinguishing between distal and proximal skin temperatures

About half of the reviewed experimental studies measured skin temperature in different body locations. This is in general a good practice as it allows for the detection of changes in various thermo-physiological parameters during the experiments. However, none of these studies have separately calculated distal and proximal skin temperatures. They have rather calculated a mean skin temperature based on different types of weighted formulae as it is most often the case in thermal comfort research. As we have discussed in the previous sections, the time course of distal skin temperature does not exactly follow the time course in proximal skin temperature and its fluctuation is always characterized by larger diurnal amplitudes [6,49]. In fact, hands and feet are the main sites for sensible heat loss [59]. Therefore, it is important to distinguish between distal and proximal skin temperatures when calculating and reporting skin temperatures for both experimental and observational studies.

## Conclusions

In this paper we reviewed 21 studies (both observational and experimental) investigating diurnal variations of thermal perception and found some contradictory findings. The existence (or absence) of diurnal patterns in human thermal perception could not be clarified. By considering the physiological evidence on thermoregulation and circadian rhythms, we list some of the possible sources of bias for the observed inconsistencies:

- inter-individual differences in circadian phase and chronotype may have made it difficult to detect time-of-day effects on thermal perception. Exogenous differences such as those caused by shift work, irregular sleeping and eating patterns, jetlag, and irregular light exposure patterns affecting such differences should be controlled in future studies.
- only three out of the 21 reviewed studies report the characteristics of the light exposure and only in terms of intensity (lux). The results of these studies could have therefore been systematically compromised by the effect of light. In future experimental studies the light exposure should be systematically controlled as part of the experimental design in terms of both intensity and spectrum, while observational studies should include the intensity and spectrum of the light as a measured variable;
- the menstrual cycle of fertile women may have at least partially masked the effect of time of day and, thus, should be included as a controlled variable in future experimental and observational studies;
- in addition to the studied thermoneutral conditions, future experimental studies should include thermal conditions which are far from neutrality in order to test whether warm and cold sensation thresholds are affected by the time of day;
- it seems particularly important to focus in the future on the circadian transition periods, morning and evening, and to compare them with the middle of the day where relatively little seems to happen in terms of circadian thermo-physiological variations;
- it is important to distinguish between distal and proximal skin temperatures when calculating and reporting skin temperatures for both experimental and observational studies, this has not been done in the reviewed studies.

Ultimately, in order to definitively answer the question of whether time-of-day affects human thermal perception and to disentangle circadian effects from other time-varying factors which might influence the daily course of thermal preferences, such as time-varying levels of activity, adequately powered experiments in highly-controlled laboratory conditions using constant routine or forced desynchrony protocols are needed.
